# Supplementary material for: Flavonoid Derivatives as Potential Cholinesterase Inhibitors in Scopolamine-Induced Amnesic Mice: An In Vitro, In Vivo and Integrated Computational Approach
Source: Brain Sci. 2022 Jun 2;12(6):731. doi: 10.3390/brainsci12060731 (PMC9221371; doi:10.3390/brainsci12060731)
Supplement: Supplementary file 1 [file brainsci-12-00731-s001.zip › brainsci-1720927-supplementary.pdf]

Article

Flavonoid Derivatives as Potential Cholinesterase Inhibitors in Scopolamine Induced Amnesic Mice; An *in vitro*, *in-vivo* and Integrated Computational Approach

Fakhria A. Al-Joufi [1], Syed Wadood Ali Shah [2], Mohammad Shoaib [2], Mehreen Ghias [2], Shafiullah [2], Atif Ali Khan Khalil [3], Syed Babar Jamal [3], Syed Muhammad Hassan Shah [4] and Muhammad Zahoor [5,*]

[1] Department of Pharmacology, College of Pharmacy, Jouf University, 72341 Aljouf, Saudi Arabia; faaljoufi@ju.edu.sa
[2] Department of Pharmacy, University of Malakand, Dir (Lower), Chakdara 18800, Khyber Pakhtunkhwa, Pakistan; pharmacistsyed@gmail.com (S.W.A.S.); mohammadshoaib13@yahoo.com (M.S.); mehreenghias@yahoo.com (M.G.); shafi_ullah34@yahoo.com (S.)
[3] Department of Biological Sciences, National University of Medical Sciences, Rawalpindi 46000, Pakistan; atif.ali@numspak.edu.pk (A.A.K.K.); babar.jamal@numspak.edu.pk (S.B.J.)
[4] Department of Pharmacy, Sarhad University of Science and Information Technology, Peshawar, 25000, Khyber Pakhtunkhwa, Pakistan; syedhassan41@gmail.com
[5] Department of Biochemistry, University of Malakand, Dir (Lower), Chakdara 18800, Khyber Pakhtunkhwa, Pakistan
* Corresponding author: mohammadzahoorus@yahoo.com

Supplementary file

Table S1. Selection of doses for pharmacological investigation using preliminary results in behavioral Y-maze test.

Treatment/Dose (mg)		Spontaneous alternation performance
Normal control		81.34±2.01
Amnesic control (Scopolamine)		36.91±1.75###
F1	2.5	37.02±2.11
	5	38.91±1.67
	7.5	49.12±1.45*
	10	48.66±1.98*
	12.5	48.98±2.01*
	15	51.72±1.88**
	20	52.03±2.03**
F5	2.5	38.63±1.78
	5	43.87±1.94
	7.5	66.53±1.89**
	10	66.97±1.72**
	12.5	67.33±2.05***

	**15**	72.94±1.89***
	**20**	73.08±2.12***

Mean±SEM (n=8). Statistically analyzed with oneway ANOVA followed by Dunnett's post hoc multiple comparison test to determine the values of P. $^{###}$P<0.001 comparison of amnesic (scopolamine) group *vs.* normal control group. *P<0.05, **P<0.01 and ***P<0.001 as comparison treated groups *vs.* amnesic group (scopolamine) using one way ANOVA followed by Dunnett's comparison.
